# Allergen exposure chambers: implementation in clinical trials in allergen immunotherapy

**DOI:** 10.1186/s13601-020-00336-9

**Published:** 2020-07-29

**Authors:** O. Pfaar, P. Zieglmayer

**Affiliations:** 1 Department of Otorhinolaryngology, Head and Neck Surgery, Section of Rhinology and Allergy, University Hospital Marburg, Philipps-Universität Marburg, Marburg, Germany; 2 Vienna Challenge Chamber, Vienna, Austria

**Keywords:** Allergen exposure chamber, Allergic, Clinical trials, Allergen immunotherapy, Phase II

## Abstract

Allergen exposure chambers (AECs) have been developed for controlled allergen challenges of allergic patients mimicking natural exposure. As such, these facilities have been utilized e.g., for proof of concept, dose finding or the demonstration of onset of action and treatment effect sizes of antiallergic medication. Moreover, clinical effects of and immunological mechanisms in allergen immunotherapy (AIT) have been investigated in AECs. In Europe AIT products have to fulfill regulatory requirements for obtaining market authorization through Phase I to III clinical trials. Multiple Phase II (dose-range-finding or proof-of-concept) trials on AIT products have been performed in AECs. However, they are not accepted by regulatory bodies for pivotal (Phase III) trials and a more thorough technical and clinical validation is requested. Recently, a Position Paper of the European Academy of Allergy and Clinical Immunology (EAACI) has outlined unmet needs in further development of AECs. The following review aims to address some of these needs on the basis of recently published data in the first part, whereas the second part overviews published examples of most relevant Phase II trials in AIT performed in AEC facilities.

## Introduction

Allergen immunotherapy (AIT) has been used for the (causal) treatment of patients with IgE-mediated allergies for more than 100 years [1] and has been demonstrated to be efficious and safe for both application forms, sublingual (SLIT) and subcutaneous (SCIT) [2–4] as recently published in systematic reviews and metaanalyses of the European Academy of Allergy and Clinical Immunology (EAACI) [5, 6]. As only disease modifying treatment option for allergic patients, evidence for its preventive capacities and long-term efficacy has been reported [7]. Furthermore, several innovations for AIT such as e.g. personalized medicine or biomarkers in AIT are currently followed [8–10] and treatment algorithms for AIT in routine care have been recently presented [11].

For gaining marketing authorisation in Europe, clinical trials for AIT products have to align with the guideline on the “*Clinical Development of Products for Specific Immunotherapy for the Treatment of Allergic Diseases*” of the European Medicines Agency (EMA) [12]. For Phase II (dose-finding and proof-of-concept) trials the EMA accepts allergen provocation tests such as conjunctival, nasal or bronchial challenge tests or challenges under standardized conditions in allergen exposure chambers (AEC) for the analysis of the primary endpoint [12–14]. Therefore, multiple phase II-trials have been performed and published in the field of AIT [15].

An early study published investigated the clinical and immunological effects of a short course sublingual birch pollen extract [16]. In this randomized double-blind, placebo-controlled parallel group trial volunteers were evaluated in terms of clinical reactivity measured by subjective and objective symptom assessment ahead and after a 3 month preseasonal treatment. Treatment effects were determined by titrated skin prick test, conjunctival provocation test and subjective and objective symptoms in an AEC. Interestingly, volunteers had to show not only positive birch pollen specific skin prick test reactivity, but also clinical reactivity proven by topical conjunctival and nasal provocation test ahead of randomization. Several phase II trials in the field of AIT followed and are reported more detailed in the following.

However, the EMA also states that for pivotal phase III trials in AIT, AECs “deemed to be a promising tool for the evaluation of efficacy”, but further clinical validation is urgently needed [12]. To address this important unmet need, the EAACI has formed a task force initiative and published a Position Paper aimed to internationally harmonize current concepts in AECs and to enhance their broader development for future clinical trials [17]. The panel of experts has outlined suggestions for procedures for the technical and clinical validation processes to fulfill the regulatory prerequisites, but also has indicated important gaps and unmet needs. Another recently published expert report on current concepts and future needs in AIT trial designs underlined the importance of further validation of AECs with regards to natural exposure [10, 18] and also their potential for pediatric trials and the requirements of the pediatric-investigational plan of the EMA [19].

The following review aims to address some of these needs on the basis of recently published data in the first part, whereas the second part overviews published examples of most relevant AIT trials in which clinical and immunological outcomes have been analysed in AEC facilities.

## Variables to be determined for efficacy analysis

Irrespective of technical and clinical validation of the respective allergen exposure chamber model there are several factors to be taken into account when developing a protocol for an efficacy study conducted with use of an AEC. In contrast to field-studies, where parameters of the natural environmental exposure of the individual subject cannot be influenced neither sufficiently determined, an AEC mimicking natural exposure conditions is nevertheless an artificial set up. Defining outcome parameters to be evaluated during the study requires to rule out the major factors influencing clinical results of the evaluation first. As a consequence, variables for efficacy analysis in AIT trials in the (artificial) chamber exposure and their clinical relevance for a real life setting (under natural environmental exposure) should be further investigated and understood.

### How does allergen exposure influence effect size?

Today sufficient evidence exists that treatment effects of AIT are best correlated with symptom severity of the patients, and evaluations of naturally exposed patients in field trials do often show less efficacy [20–22]. Limiting factors for the demonstration of clinical efficacy usually are e.g., the fluctuation of natural pollen exposure during pollen seasons and corresponding symptom severity of the patients, retrospective symptom scoring for baseline evaluation and the combined evaluation of patients with different symptom severity and others [15, 23]. Taken together, the relevant exposure of the individual patient in field trials is very heterogeneous, overall unpredictable and variable due to climatic and meteorological reasons. In general, the relevance of this methodological problem should be described and emphasized more clearly and solutions should be elaborated. This aspect underlines the potential of AEC facilities for future trials in AIT.

### Which parameters reflect clinical efficacy?

To avoid production of unfeasible data during a clinical trial there would be some restrictions to select patients suitable for allergen challenge trials: skin prick test results do not necessarily correlate with clinical reactivity, therefore one important inclusion criterion for an AEC trial should be proven clinical reactivity during challenge session. Recent data on correlation between skin test reactivity and clinical rhinitis symptoms are heterogenous: In a study published by Ellis et al. the investigation of 123 patients with ragweed pollen allergy showed no correlation between skin prick test reactivity and clinical rhinitis symptoms [24]. A trial of Huss-Marp and colleagues determining the correlation between different challenge tests, skin prick test reactivity and specific IgE-levels in 104 grass pollen allergic patients revealed a correlation between skin test reactivity and sIgE-levels, but not of clinical reactivity and specific IgE-levels [25]. Several years ago it already had been shown [26] on molecular level that skin test positivity, but not immunoglobulins, can be a sensitive marker for clinical reactivity, but several immunological variables have to be considered: grass and birch pollen allergics were evaluated regarding their IgE-, IgG-, skin test and nasal provocation test (NPT) reactivity on purified recombinant Phl p 1, Phl p2, Phl p5, Bet v1 and Bet v2. Patients showed a differentiated sensitization profile and no correlation was evident neither between sIgE-level and clinical reactivity (r = 0.2) or wheal diameter (r = 0.1). Allergenic potency was even higher in minor allergens than in major allergens, which was reflected by a high correlation (r = 0.63, p > 0.01) of wheal diameter and clinical reactivity; IgG levels and function were irrelevant for any of the outcome parameters determined.

Of increasing relevance also for practice routine use is conjunctival provocation testing (CPT), as it had been shown already several years ago that CPT is a suitable measure for clinical reactivity even in children with allergic rhinitis not suffering from conjunctival symptoms [27]. A large body of evidence has been generated by Mösges et al. [28, 29] proving the suitability of standardized CPT protocols for the assessment of efficacy of immunotherapy. However, the conclusion drawn from the most recent review [30] that CPT works in 3 out of 4 studies may be a result of the fact that extracted instead of natural crude allergenic materials must be used for topical allergen provocation tests not always reflecting the individual sensitization profile of the patient and the natural environmental exposure conditions, respectively. The same seems to be true for nasal provocation tests with highly heterogenic evidence in the literature regarding NPT as assessment tool for clinical reactivity in general [31] or correlation with reactivity in allergen challenge chamber exposure [32]. These results suggest that sensitization profiles of patients are as diverse as allergenic materials used for testing and until today a distinctive (clinical and non-clinical) parameter as relevant endpoint in reflecting clinical efficacy in AIT trials has not been established. So further research on AEC facilities may address this important need.

### Can we detect priming effects?

AEC trials do not provide long term evaluation with induction and direct evaluation of late phase reactions, but assessment of priming effects whether environmental or induced by pre-challenges is possible within AEC trials: Differences in clinical reactivity immediately after season and out of season, corresponding to a priming effect, were investigated by Yuki et al. [33]. In the present trial, the authors stated that symptoms were induced more rapidly after the end of pollen season than with out-of-season exposure. During pollen season with variable amounts of pollen counts patients often exhibit symptom-free intervals during phases of low pollen exposure. But nevertheless upregulation of inflammatory cells and mediators occur. This minimal persistent inflammation leads to increased sensitivity and inflammatory response to allergens. This evidence is not new, as quantitative assessment of the impact of intercurrent perennial allergies on the clinical reactivity in pollen allergies had been determined already 20 years ago, when Toth et al. were able to show a faster and more pronounced allergic reaction on grass pollen exposure in the Vienna Challenge Chamber in patients with intercurrent cat and dog allergies [34] Recently is has been shown by North et al. [35], that epigenetic changes become evident within 3 h of allergen exposure. The investigators exposed grass pollen allergic rhinitis sufferers on 2 consecutive days to grass pollen in an AEC for 3 h each and were able to detect immediate adaptive DNA methylation patterns, which also reflect clinical reactivity of the patients. The result of such a priming effect is a more rapid onset of symptoms, but not a higher extent of symptoms, as shown by Yuki et al. [33]. That priming effects do not necessarily influence the overall amount of symptoms, could be demonstrated in several other studies as well. The effect of a combination of 10 mg Cetirizine and 120 mg Pseudoephedrine was investigated by Badorrek et al. [36] in a randomized, placebo-controlled, double-blind, four-way crossover study in 70 patients with grass pollen allergy. The preparation, which is established since the 1990s, was administered 2 h after start of each 6 h AEC session. In this study, there was no difference in clinical efficacy of the preparation, unless given in seasonally primed patients or outside the season. Priming effects induced by AEC sessions can be quantified in e.g. onset and duration of action protocols to evaluate the 24-h coverage of a once daily antihistamine. In double blind-placebo controlled (DBPC) cross-over studies in the Vienna Challenge Chamber allergic volunteers were exposed for 4 h and treated 2 h after start, when they had developed hay fever symptoms. During the first 2 h after treatment the onset of action was determined for the active compounds vs. placebo. 22 h after drug intake volunteers were re-exposed to quantify the residual efficacy and it could be demonstrated repeatedly that the primed trial participants exhibited an accelerated onset, but not an aggravated level of symptoms [37, 38]. On the other hand some study data suggest the utilization of priming measures to induce adequate allergic symptoms in low sensitized patients. Jacobs et al. [39] determined in their mountain cedar validation study different levels of clinical reactivity in non-, mild to moderate and highly sensitized patients and could demonstrate that priming subjects over a short time period of 2 h is more effective in inducing symptoms than a long term exposure of 5 h in an allergen exposure chamber. The current evidence indicates that on the one hand AEC trials can be conducted not only outside the pollen season, but also inside the pollen season, as the amount of symptoms is not necessarily influenced by environmental allergen exposure. But on the other hand standardized priming measures can be utilized to induce a symptom level required to adequately assess treatment effects. Though more trials are needed to confirm these findings, the results indicate that the performance of AEC trials for therapeutic interventions such as e.g. AIT are not limited to certain seasons of the year.

### Use of different AEC allergen models in AIT trials

The use of AECs as outcome measure for assessment of clinical efficacy of different immunotherapeutic preparations is still limited by the operational availability of validated systems and allergen models on the one hand and by regulatory restrictions on the other hand. Most established AEC allergen models used for IT trials are grass pollen and house dust mite, as for these predominant target allergen sources properly validated systems [40, 41] and study designs are available. The following will overview representative examples of different clinical trials on AIT which have analysed clinical and immunological effects as primary or secondary endpoints in AEC facilities:

#### i. Grass pollen AIT trials

**Fig. 1:**
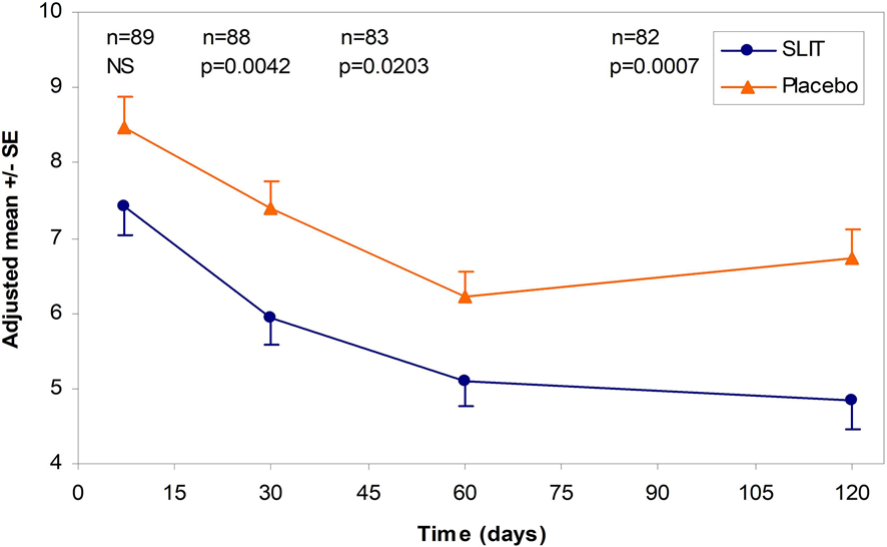
Average RTSS at each challenge during the treatment period (after 1 week, 1 month, 2 months and 4 months of treatment): the means were adjusted for the baseline covariate ± standard error (SE); reproduced from [42] with kind permission from Elsevier

In the first published grass pollen IT AEC study the onset and clinical efficacy of a sublingual grass pollen tablet formulation was investigated in 89 allergic rhinitis patients [42]. Patients were randomized to receive a 300IR active or placebo tablet and were assessed in the Vienna Challenge Chamber at baseline, after 1 week, 1 month, 2 months and 4 months of treatment. First treatment effects on nasal and ocular symptoms were detectable after 1 week, statistically significant efficacy could be shown after 1 month (Fig. 1). Furthermore the preparation was extensively investigated in terms of immunological changes and potential biomarkers [43–46]. These data largely contributed to our current knowledge in the field of biomarker assessment.

An innovative approach published 2016 was the first to investigate the effects of vaccination with a B cell epitope-based recombinant allergy vaccine [47]. The vaccine contains recombinant fusion proteins consisting of allergen-derived peptides bound to a hepatitis B virus surface antigen as immunological carrier. 70 patients were treated with 3 different doses and placebo and evaluated in the Vienna Challenge Chamber ahead and after treatment. Clinical endpoints assessed were TNSS, TOSS and SPT reactivity. It could be demonstrated that three injections of the recombinant B cell epitope-based allergy vaccine were well tolerated and effective in the middle of the three different doses with no further effect of the higher dose.

**Fig. 2:**
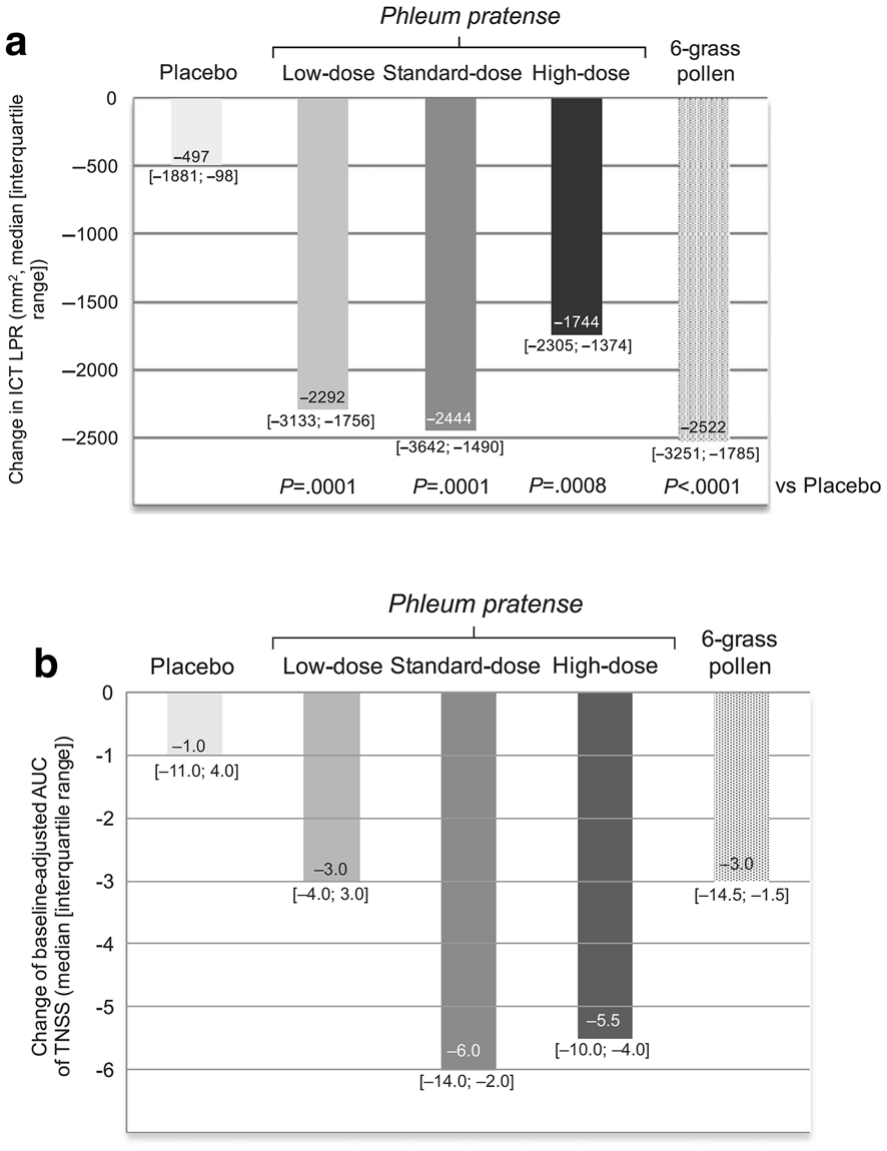
Change in intracutaneous test (ICT) late phase reaction (**a**) and of baseline- adjusted area under the curve (AUC) of Total Nasal Symptom Score (TNSS) (**b**); reproduced from [48] with kind permission from Wiley

In 2017 study data of two different formulations were published: Pfaar et al. [48] investigated three different doses of a Phleum pratense allergoid for subcutaneous application. In this five arm study the target dose (6.000TU), one fifth (1.800TU) and the threefold dose (18.000TU) of the preparation were compared to the marketed 6-grass allergoid of the same manufacturer and placebo in 102 patients. Patients were evaluated ahead and after a 4 month treatment course consisting of seven updosing and two maintenance dose injections. Clinical endpoints assessed were late phase reaction (LPR) of intracutaneous test (ICT) and total nasal symptom score (TNSS) during a 2 h session in the AEC of the Fraunhofer Institute ITEM (Fig. 2). The largest treatment effect was shown for the standard dose of 6.000TU with no further increase in the high dose-group.

In a study performed by Ellis et al. [49] it could be demonstrated, that a grass pollen epitope peptide, applied intradermally, is most effective in terms of rhinoconjunctivitis total symptom score, when given in lower doses, but more frequently and in shorter intervals, than in higher doses, given less frequently and in longer intervals. Higher symptomatic patients showed better efficacy of the effective dosing regimen compared to less symptomatic patients when evaluated in an allergen exposure chamber repeatedly over 4 consecutive days.

#### ii. Mite AIT trials

**Fig. 3:**
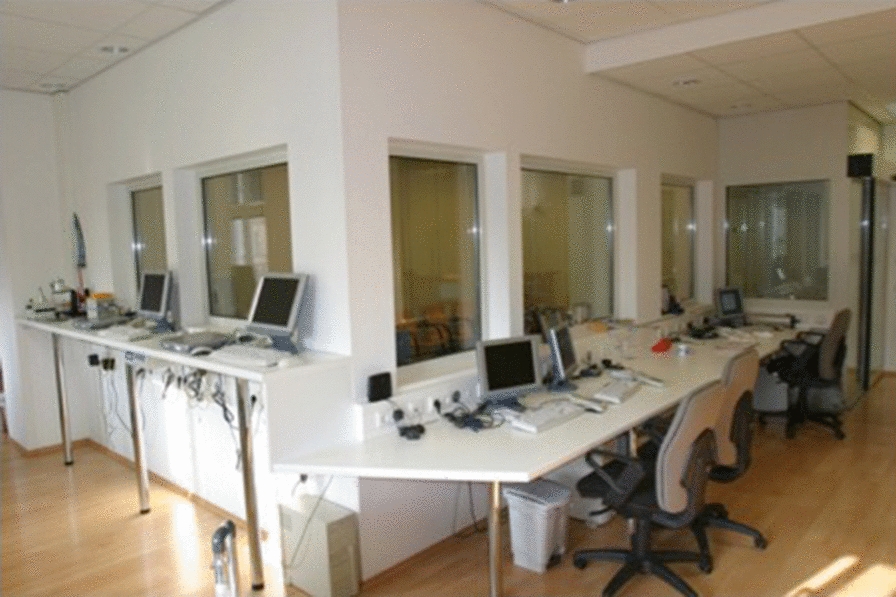
Vienna challenge chamber

**Fig. 4:**
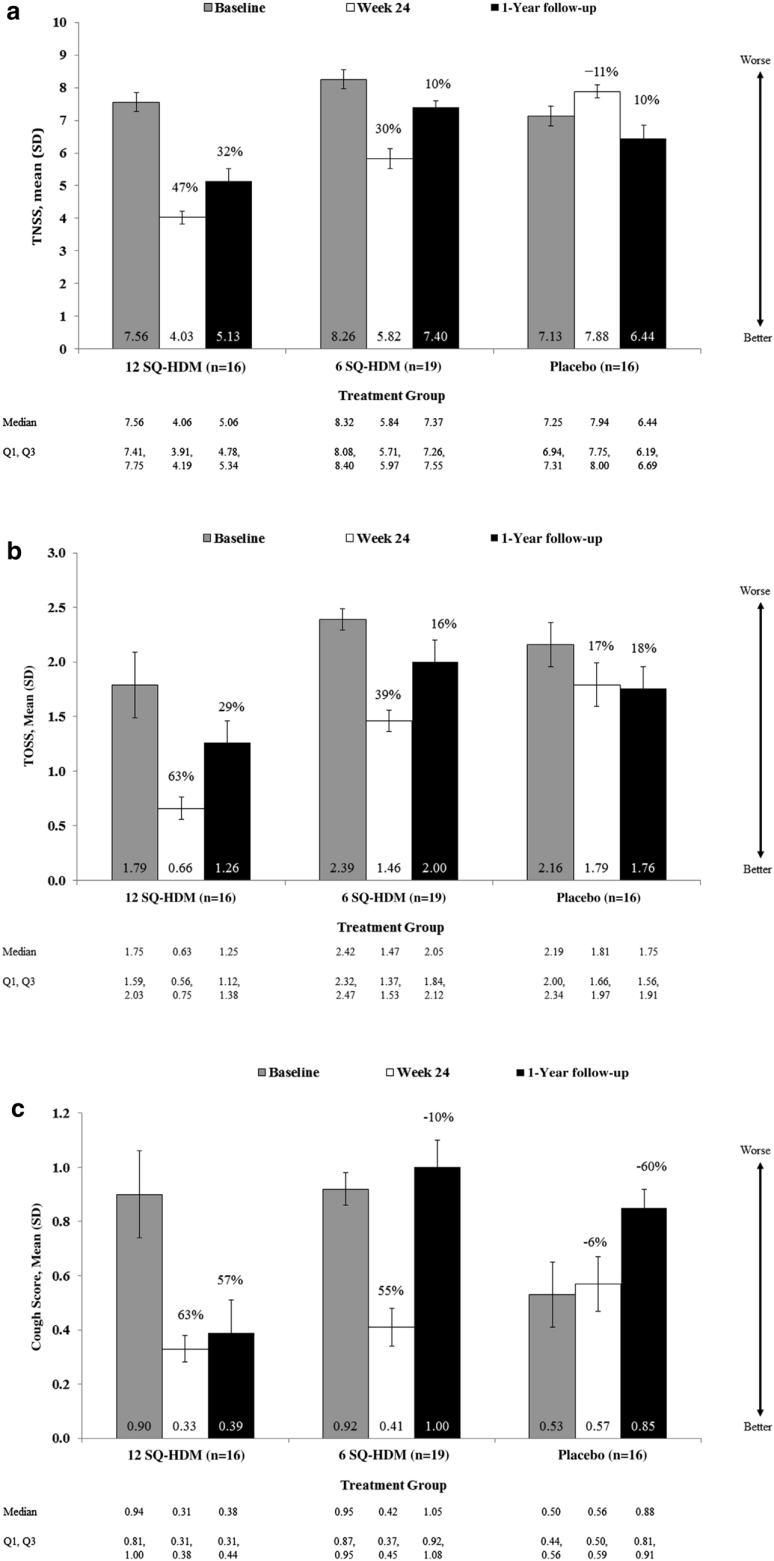
**a** TNSS, **b** TOSS and **c** cough score at baseline, week 24, and 1-year follow-up (all participants with follow up). Percentages represent improvements relative to baseline; reproduced from [51] with kind permission from Elsevier

Until today two different mite preparations were evaluated within AEC assessments. Both are mite extract tablet formulations for sublingual application. One preparation was evaluated in terms of dose finding, onset of action, immediate and long term efficacy in the Vienna Challenge Chamber (Fig. 3) [50, 51]. 124 patients were treated with 3 different doses versus placebo for 6 months and were assessed in the Vienna Challenge Chamber ahead, after 2, 4 and 6 months of treatment in terms of TNSS. A dose- and time-dependent effect of the SLIT tablet on nasal, ocular and asthma symptoms was observed, with maximum efficacy of the highest dose at week 24 after 6 months of treatment. It could be shown that higher symptomatic asthmatic patients benefit more from the active treatment. A subset of patients (n = 51) could be reevaluated 1 year after cessation of treatment. A sustained improvement of symptoms was still evident in the high dose group (Fig. 4).

Another large scale study (n = 355) investigated 3 different doses versus placebo of another mite extract tablet formulation in an AEC [52]. Patients assessed their rhinitis total symptom score (RTSS) during a 4 h challenge session in Ontario, Canada, ahead, after 1,2,4 and 6 months of treatment. A dose-dependent effect was determined after 6 months of treatment compared to baseline.

#### iii. Cat AIT trials

Clinical efficacy of intracutaneous Fel d 1 derived peptides was determined with help of a challenge chamber model [53]. This study was a randomized, double-blind, placebo-controlled, parallel-group study evaluating the safety and efficacy of intradermal injections of 2 dosing regimens of Cat-PAD performed in Canada. The research group used a validated allergen dispensing system to ensure a mean airborne level of around 50 ng of Fel d 1/m3. The authors determined the chosen allergen amount to be comparable to previously measured home exposure levels of 10 to 200 ng/m^3^ in homes with cats. 202 subjects were treated with the peptide or placebo and were exposed to cat allergen ahead of the treatment for baseline evaluation and around 5 months after start of treatment. Patients underwent 3 h challenges on 4 consecutive days in the ACC. A subgroup of 89 patients was followed up and rechallenged 1 year after start of treatment. A significantly better efficacy was shown with 4 administrations separated by 4 weeks of the 6 nmol-dose than for 8 administrations separated by 2 weeks of the 3 nmol-dose of the preparation.

#### iv. Birch pollen AIT trials

The optimal dose of a synthetic vaccine consisting of a Bet v 1 folding variant for subcutaneous application was evaluated in 37 birch pollen patients in the AEC of the Fraunhofer Institute ITEM [54]. Patients were treated with 4 different doses of the vaccine (20 µg, 80 µg, 160 µg, 320 µg) or placebo for 10 weeks. Clinical efficacy was determined by 8 h birch pollen challenge sessions ahead and after treatment.

Clinical efficacy was assessed by intracutaneous test and different symptom scores during allergen challenge sessions and 80 µg turned out to be the dose with the best risk/benefit ratio.

#### v. Ragweed pollen AIT trials

Until today one study is published using a ragweed AEC model for assessment of a ragweed IT preparation [55]. In the respective study a ragweed allergoid was applied to 228 patients subcutaneously 4 times in weekly intervals, and clinical efficacy was determined by TNSS and TNNSS during 3 h allergen challenge sessions on 4 consecutive days ahead and after treatment. Difference in terms of TSS (sum of TNSS and TNNSS) was 25% between active and placebo group after treatment in favor of the active treatment.

## Conclusions

Allergen Exposure Chambers (AECs) have been developed and further optimized throughout the recent years. In the field of allergen immunotherapy (AIT) different AEC allergen models have been broadly utilized in Phase II trials with some of them outlined more in detail in this article. Beneath this, further technical and clinical validation has been strongly recommended by both regulatory authorities and academical societies. Firstly, variables for efficacy analysis in AIT trials in the (artificial) chamber exposure and their clinical relevance for a real life setting (under natural environmental exposure) should be further investigated and understood. Secondly, as the relevant exposure of patients in field trials is very heterogeneous, overall unpredictable and variable due to climatic and meteorological reasons, the relevance of this methodological problem should be described and emphasized more clearly, and solutions should be elaborated. A more broader utilization of AEC facilities for future trials in AIT may bypass this. In this context, further research should focus on (clinical and non-clinical) parameters as relevant endpoints in reflecting clinical efficacy of AIT also considering molecular aspects. A better understanding about similarities and differences in technical specification on AEC facilities may address this important need and is currently in the focus of a Task Force of the European Academy of Allergy and Clinical Immunology as a first step of further evaluation and validation of AEC facilities. Recent data indicate that seasonal priming does not significantly impact or alter the amount of the clinical response of therapeutical interventions demonstrated in co-seasonal AEC sessions compared to AEC challenges out of the pollen season. Though more trials are needed to confirm these findings, the results indicate that the performance of AEC trials for therapeutic interventions such as e.g. AIT are not limited to certain seasons of the year. A Task Force initiative of the European Academy of Allergy and Clinical Immunology is in the process of further evaluation and reporting of potential of AEC for the field of AIT development.

## Data Availability

Not applicable (review-article).

## Abbreviations

AEC: Allergen exposure chamber
AIT: Allergen immunotherapy
ARTSS: Average rhinoconjunctivitis total symptom score
BC: B cell
CPT: Conjunctival provocation test
EAACI: European Academy of Allergy and Clinical Immunology
EMA: European Medicines Agency
HDM: House dust mite
ICT: Intracutaneous test
LPR: Late phase reaction
NPT: Nasal provocation test
RMM: Rhinomanometry
SLIT: Sublingual immunotherapy
SPT: Skin prick test
TASS: Total asthma symptom score
TC: T cell
TNSS: Total nasal symptom score
TNNSS: Total non-nasal symptom score
TOSS: Total ocular symptom score
TRSS: Total rhinitis symptom score
